# An organotypic human melanoma-in-skin model as an *in vitro* tool for testing Vγ9Vδ2-T cell-based immunotherapy

**DOI:** 10.1016/j.iotech.2024.100724

**Published:** 2024-07-10

**Authors:** E. Michielon, L.A. King, T. Waaijman, M. Veth, S.W. Spiekstra, H.J. van der Vliet, S. Gibbs, T.D. de Gruijl

**Affiliations:** 1 Department of Molecular Cell Biology and Immunology, Amsterdam University Medical Center Location Vrije Universiteit Amsterdam, Amsterdam; 2 Amsterdam Institute for Infection and Immunity, Amsterdam University Medical Center, Vrije Universiteit, Amsterdam; 3 Cancer Center Amsterdam, Cancer Biology and Immunology Program, Amsterdam University Medical Center, Vrije Universiteit, Amsterdam; 4 Department of Medical Oncology, Amsterdam University Medical Center Location Vrije Universiteit Amsterdam, Amsterdam; 5 Lava Therapeutics NV, Utrecht; 6 Department of Oral Cell Biology, Academic Centre for Dentistry Amsterdam (ACTA), University of Amsterdam and Vrije Universiteit, Amsterdam, The Netherlands

**Keywords:** melanoma, immunotherapy, Vγ9Vδ2-T cells, tumor microenvironment, in vitro reconstructed human skin

## Abstract

**Background:**

Despite considerable advancements in cancer immunotherapy, advanced melanoma still presents a substantial clinical challenge. In an effort to explore treatment options, we examined the immunotherapeutic potential of effector Vγ9Vδ2-T cells *in vitro* in a three-dimensional (3D) human organotypic melanoma-in-skin (Mel-RhS) model.

**Materials and methods:**

Vγ9Vδ2-T cells were introduced into Mel-RhS via intradermal injection and cultured within the tissue microenvironment for up to 3 days.

**Results:**

Vγ9Vδ2-T cells remained viable for up to 3 days and were in close proximity to or within tumor nests. Upon Mel-RhS dissociation, a fraction was shown to be decorated by melanoma-associated chondroitin sulfate proteoglycan (MCSP), demonstrating their ability to actively navigate the tumor microenvironment and trogocytose cancer cells. Investigation into the apparent trogocytosis revealed an enhanced activated state of MCSP-decorated Vγ9Vδ2-T cells, evidenced by increased expression levels of 4-1BB, NKp44, programmed cell death protein-1 (PD-1), and programmed death-ligand 1 (PD-L1), compared with their MCSP^−^ counterpart. These findings suggest that Vγ9Vδ2-T cells, upon successfully contacting melanoma cells, actively recognize and acquire MCSP from these malignant cells. Evidence of actual tumor cell elimination, although not significant, was only obtained after preincubation of Mel-RhS with pamidronate, a phosphoantigen-inducing agent, indicating the need for additional T cell receptor-mediated signaling for Vγ9Vδ2-T cells to reach their full oncolytic potential.

**Conclusions:**

This study highlights the viability and persistence of Vγ9Vδ2-T cells within the 3D microenvironment, their migratory and antitumor functionality, and the suitability of the model for testing T cell-based therapies, contributing both to the understanding of Vγ9Vδ2-T cell biology and their application in cancer immunotherapy.

## Highlights

• Mel-RhS is suitable for testing immune cell-based therapies.
• Vγ9Vδ2-T cells survive within the tumor microenvironment for up to 3 days upon injection into Mel-RhS.
• Vγ9Vδ2-T cells migrated within the skin microenvironment toward the melanoma cells.
• MCSP acquisition reflects selectively activated Vγ9Vδ2-T cells.

## Introduction

Melanoma arises from the uncontrolled growth of melanocytes, the pigment-producing cells of the skin, and, once metastasized, is one of the most aggressive forms of cancer. Although immune checkpoint blockade has considerably improved clinical outlook, in advanced stages, more than half of patients with melanoma will still succumb to the disease.^1^ There is thus a pressing need to explore novel approaches to improve the outcome of immunotherapy for metastatic melanoma. Recently, among the various immune cells known to be implicated in antitumor responses, γδ-T cells have gained significant attention given their potent antitumor activity.^2^ Among them, Vγ9Vδ2-T cells represent the predominant subset (95%) in human peripheral blood^3^ and, unlike conventional αβ-T cells, recognize ligands independent of human leukocyte antigen (HLA) molecules.^4^^,^^5^ Vγ9Vδ2-T cells respond to nonpeptide antigens, *i.e.* phosphoantigens (pAgs), derived from the mevalonate pathway, which are upregulated in various types of cancer, including melanoma.^6^, ^7^, ^8^ Upon activation, Vγ9Vδ2-T cells rapidly expand and exert their antitumor effector functions, including direct tumor cell killing via the production of cytotoxic molecules (e.g. granzymes); secretion of proinflammatory cytokines, such as interferon-gamma and tumor necrosis factor-alpha^9^; antigen cross-presentation^10^^,^^11^; and induction of cytotoxic responses by other immune cells [e.g. dendritic cells (DCs), natural killer (NK) cells, and αβ-T cells], leading to enhanced anticancer responses.^12^ These unique properties make Vγ9Vδ2-T cells an attractive candidate for immunotherapeutic strategies aiming to control melanoma progression. Understanding the mechanisms underlying their antitumor activity and overcoming the immunosuppressive tumor microenvironment will be crucial for the development of effective Vγ9Vδ2-T cell-based therapies against melanoma. By gaining insights into these crucial aspects, we aim to enhance our understanding of the therapeutic potential of Vγ9Vδ2-T cells and thus identify strategies to optimize their efficacy in treating melanoma, ultimately improving the prognosis and quality of life for patients with cancer.

Three-dimensional (3D) *in vitro* melanoma-reconstructed human skin (Mel-RhS) models serve as an intermediate step between simplistic two-dimensional (2D) cultures and complex *in vivo* systems, providing a valuable tool for hypothesis testing, mechanistic investigations, and initial screening of therapies. However, inclusion of immune cells in such systems remains challenging and only few research groups have reported the generation of immune cell-complemented Mel-RhS.^13^^,^^14^ Here, we studied if our previously developed Mel-RhS^15^^,^^16^ could support Vγ9Vδ2-T cell survival and allowed the investigation of their functional capabilities (*i.e.* tumor infiltration and recognition, and activation) in a physiologically highly relevant *in vivo-*like setting.

## Materials and methods

### Cell isolation and culture

#### Skin cells

Human skin was obtained as surgical leftover material after obtaining informed consent from healthy donors who underwent abdominal dermolipectomy. Skin samples were used in an anonymized fashion in accordance with the ‘Human Tissue and Medical Research: Code of Conduct for Responsible Use’, as formulated by the Federation of Dutch Medical Scientific Societies (www.federa.org). Epidermal cells (keratinocytes and melanocytes) and dermal fibroblasts were isolated and cultured as previously described^17^^,^^18^ and were used up to second and third passage, respectively, in all the experiments. Epidermal cells were cultured in Dulbecco’s modified Eagle medium (DMEM; Gibco, Grand Island, NY)/Ham’s F-12 (Gibco) in a 3 : 1 ratio, 1% penicillin/streptomycin (P/S; Invitrogen, Paisley, UK), 1% Ultroser G (UG; BioSepra S.A., Cergy-Saint-Christophe, France), 0.1 μM insulin (Sigma-Aldrich, St. Louis, MO), 1 μM hydrocortisone (Sigma-Aldrich), 1 μM isoproterenol (Sigma-Aldrich), and 2 ng/ml keratinocyte growth factor (Sigma-Aldrich) at 37°C and 7.5% CO_2_. Dermal fibroblasts were cultured in DMEM, 1% P/S, and 1% UG at 37°C and 5% CO_2_.

#### Melanoma cells

The human melanoma cell line A375 (CRL-1619; ATCC, Manassas, VA) was cultured in DMEM, 10% fetal bovine serum (Corning Life Sciences, Corning, NY), and 1% P/S at 37°C and 5% CO_2_. Upon receipt from the commercial supplier, the A375 cell line was expanded and low-passage stock vials were frozen. Cells resuscitated from the original vial were used up to 10 passages and cultured for no longer than 3 months before thawing a new stock vial. The cell line was not reauthenticated but was tested and scored negative for *Mycoplasma.*

#### Vγ9Vδ2-T cells

Healthy donor-derived Vγ9Vδ2-T cells were isolated, expanded, and cultured as previously described.^19^ In brief, Vδ2^+^ cells were isolated from peripheral blood mononuclear cells (PBMCs) by magnetic-activated cell sorting using anti-T cell receptor (TCR) Vδ2-FITC monoclonal antibodies (1 : 100, clone IMMU389; Beckman Coulter, Brea, CA) in combination with anti-mouse IgG MicroBeads (Miltenyi Biotec, Bergisch Gladbach, Germany) and cultured weekly with irradiated feeder mix (50 gray) consisting of PBMCs from two healthy donors, JY cells, 10 U/ml interleukin (IL)-7 (R&D Systems, Hinnerup, Denmark), 10 ng/ml IL-15 (R&D Systems), and 50 ng/ml phytohemagglutinin; Thermo Fisher Scientific, Waltham, MA). Vγ9Vδ2-T cell purity was maintained at > 95%.

### 2D co-cultures

Vγ9Vδ2-T cells were co-cultured for 24 h with either melanoma cells, fibroblasts, or epidermal cells at an effector (E)-to-target (T) ratio of 1 : 1 in flat 96-well plates. Target cells were preincubated for 6 h in the presence or absence of 10 μM pamidronate (PAM; Sigma-Aldrich). Fibroblasts and epidermal cells were seeded into wells coated with 2 mg/ml fibrin and 0.5 μg/cm^2^ collagen IV (Sigma-Aldrich), respectively. The medium consisted of 2-[4-(2-hydroxyethyl)piperazin-1-yl]ethanesulfonic acid (HEPES)-containing Roswell Park Memorial Institute (RPMI) medium (Gibco), 100 IU/ml sodium penicillin (Life Technologies, Carlsbad, CA), 100 μg/ml streptomycin sulfate (Life Technologies), 2 mM l-glutamine (Life Technologies), β-mercaptoethanol (Merck, Darmstadt, Germany), and 10% fetal calf serum (HyClone, GE Healthcare, Chicago, IL), referred to as RPMI^+++^. Cells were collected and washed with phosphate-buffered saline (PBS), followed by flow cytometry analysis.

### Reconstructed human skin cultures with or without melanoma cells

**Figure 1:**
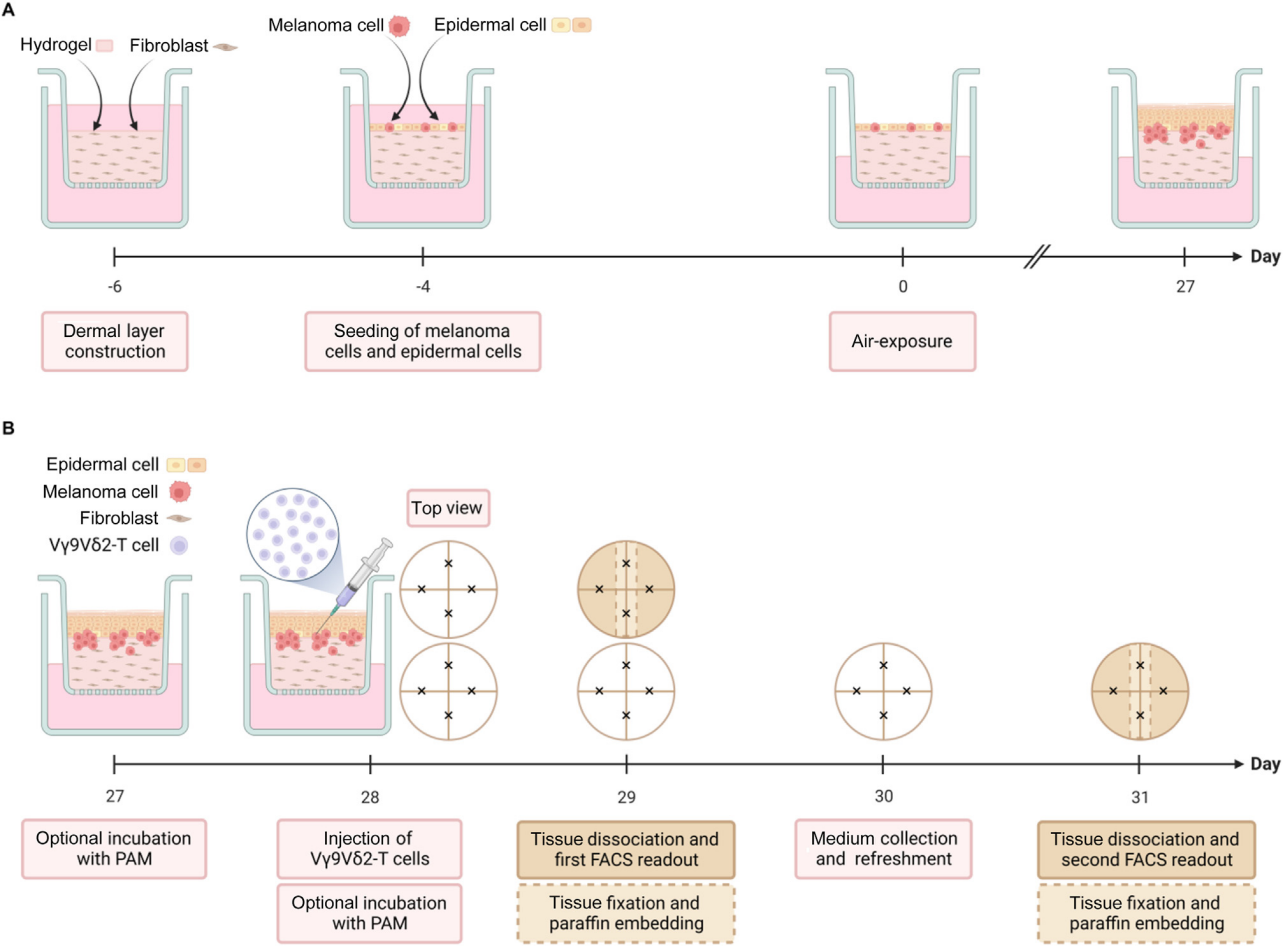
**Overview of the development of Mel-RhS and of the injection of Vγ9Vδ2-T cells into the 3D model.** (A) The dermal layer was constructed by mixing primary human dermal fibroblasts with a 1 : 1 fibrinogen/collagen hydrogel. After 2 days, A375 melanoma cells were seeded onto the reconstructed dermal compartment 2 h before epidermal cell seeding. Constructs (Mel-RhS) were then cultured for 4 days in submerged conditions and were subsequently exposed to the air–liquid interface for 27 days. RhS were constructed by omitting the melanoma cell seeding. (B) Vγ9Vδ2-T cell injection procedure and downstream analyses of the injected RhS and Mel-RhS (Mel-RhS is shown as an example). RhS and Mel-RhS were preincubated in the absence or presence of pamidronate (PAM) for 1 day. Vγ9Vδ2-T cells were then injected in four spots, indicated by black crosses, into 27-day-air-exposed RhS and Mel-RhS models, whose top view is represented as a circle (PAM was added again to those cultures which were preincubated with it). After 1 or 3 days, experiments were interrupted: a part of tissue (beige fill) was cut for conventional paraffin embedding, while the rest (brown fill) was dissociated and the resulting single-cell suspension was analyzed by flow cytometry. Created with BioRender.com. FACS, fluorescence-activated cell sorting.

RhS and Mel-RhS were constructed essentially as previously described.^15^^,^^16^ A schematic overview is displayed in Figure 1A. In brief, Mel-RhS models were created by seeding 2.5 × 10^4^ melanoma cells onto the dermal compartment 2 h before epidermal cell seeding. RhS and Mel-RhS were cultured at the air–liquid interface in DMEM/Ham’s F-12 (3 : 1), 1% P/S, 1 μM isoproterenol, 0.1 μM insulin, 2 ng/ml keratinocyte growth factor, 1 ng/ml epidermal growth factor (Sigma-Aldrich), 10 mM l-serine (Sigma-Aldrich), 10 μM l-carnitine (Sigma-Aldrich), 25 μM palmitic acid (Sigma-Aldrich), 7 μM arachidonic acid (Sigma-Aldrich), 15 μM linoleic acid (Sigma-Aldrich), 0.4 mM ascorbic acid (Sigma-Aldrich), 1 μM vitamin E (Sigma-Aldrich), referred to as KCII medium, and 0.5 μM hydrocortisone. Medium was refreshed two times a week.

### Vγ9Vδ2-T cell injection into RhS and Mel-RhS

An overview of the injection process and downstream analyses is presented in Figure 1B. One day before Vγ9Vδ2-T cell injection, RhS and Mel-RhS were incubated in the presence or absence of 20 μM PAM in the culture medium. Vγ9Vδ2-T cells were separated from the feeder mix by density gradient using Lymphoprep (STEMCELL Technologies, Vancouver, BC, Canada) and resuspended in RPMI^+++^ with 10 ng/ml IL-15 at a concentration of 20 × 10^6^ cells/ml. RhS and Mel-RhS were removed from transwells and placed onto a lid of a sterile Petri dish. Four injections of 10 μl of Vγ9Vδ2-T cell suspension each were applied per *in vitro* model using micro-fine insulin syringes (BD Biosciences, Franklin Lakes, NJ). Injected RhS and Mel-RhS were placed back in their respective transwell in 1.5 ml of KCII medium with 5 U/ml IL-7 and 5 ng/ml IL-15. PAM (20 μM) was added again to these RhS and Mel-RhS which were preincubated with it the day before. As controls, RhS and Mel-RhS were injected following the same procedure with RPMI^+++^ containing 10 ng/ml IL-15 (mock injection). After 1 or 3 days, part of the RhS or Mel-RhS was cut out and fixed overnight in 4% formaldehyde (VWR, Radnor, PA) and prepared for immunohistochemical analysis, while the rest was processed for tissue dissociation and single-cell preparation.

### Tissue dissociation to single-cell suspensions

RhS and Mel-RhS were cut into small pieces with a surgical blade, resuspended in Iscove modified Dulbecco medium (Gibco), 0.1% DNAse I (Roche, Basel, Switzerland), 0.14% Collagenase A (Roche), 100 IU/ml sodium penicillin, 100 μg/ml streptomycin sulfate, 2 mM l-glutamine, 25% dispase II (Roche), and 5% fetal calf serum, transferred to sterile flasks, and incubated on a magnetic stirrer for 45 min at 37°C. After incubation, cell suspensions were run through a 100 μM cell strainer and washed with PBS.

### Flow cytometry

Cells from the 2D co-cultures or from dissociated RhS and Mel-RhS were stained for 30 min at 4°C with a mixture of the following monoclonal antibodies: anti-CD3-APC-Cy7 (1 : 25, clone SK7; BD Biosciences), anti-TCR Vδ2-BV711, anti-TCR Vγ9-BV510 (1 : 50, clone B3; BD Biosciences), anti-melanoma-associated chondroitin sulfate proteoglycan (MCSP)-APC (1 : 50, clone LHM-2; R&D Systems), anti-4-1BB-PE (1 : 50, clone 4B4-1; BD Biosciences), anti-NKp44-PE-Cy7 (1 : 40, clone REA1163; Miltenyi Biotec), anti-programmed cell death protein-1 (PD-1)-BV786 (1 : 20, clone EH12.1; BD Biosciences), anti-programmed death-ligand 1 (PD-L1)-FITC (1 : 75, clone MIH2; BioLegend), and anti-BTN3A-PE (1 : 50, clone BT3.1; Miltenyi Biotec). Before acquisition, 7-AAD and 123count eBeads (Thermo Fisher Scientific) were added to the cell pellet to determine cell viability and absolute cell numbers. Cells were measured on the LSRFortessa XL-20 (BD Biosciences) and data were analyzed using FlowJo version 10.7.2 (BD Biosciences).

### Immunohistochemistry and immunofluorescence

Formalin-fixed paraffin-embedded 5-μm-thick tissue sections were stained for CD3 (1 : 100, M7254, clone F7.2.38; Dako, Glostrup, Denmark). Sections were immersed in 10 mM Tris/1 mM ethylenediaminetetraacetic acid (EDTA) buffer (pH 9.0) for 15 min at 100°C, followed by slowly cooling down to room temperature (RT), washed with PBS, and incubated with the primary antibody for 1 h at RT, followed by incubation with BrightVision plus poly-HRP-anti-mouse/rabbit IgG (Immunologic, VWR) and 3-amino-9-ethylcarbazole (Sigma-Aldrich) substrate, and by a counterstain with hematoxylin. Stained tissue sections were photographed using the VS200 slide scanner (Olympus, Tokyo, Japan). Alternatively, formalin-fixed paraffin-embedded 5-μm-thick tissue sections were co-stained for CD3 (1 : 100, A0452; Dako) and granzyme B (1 : 300, clone GrB-7, MON7029C; Monosan, Uden, The Netherlands). Sections were processed for endogenous peroxidase blocking by incubation with 0.3% (v/v) H_2_O_2_ in methanol for 30 min, immersed in 10 mM Tris/1 mM EDTA buffer (pH 9.0) for 15 min at 100°C, followed by slowly cooling down to RT, washed with PBS, and incubated with the primary antibodies for 1 h at RT. Sections were then incubated with goat anti-mouse IgG Alexa Fluor 555 (1 : 200, A28180; Thermo Fisher Scientific) and goat anti-rabbit Alexa Fluor 647 (1 : 200, A21245; Thermo Fisher Scientific) secondary antibodies for 30 min at RT. A mounting medium containing 4′,6-diamidino-2-phenylindole (DAPI; Abcam, Cambridge, UK) was used to mount coverslips. Stained tissue sections were photographed using the Vectra Polaris automatic imaging system (Akoya Biosciences, Marlborough, MA).

### Statistical analysis

Statistical analysis was carried out using GraphPad Prism 9 software version 9.5.1 (GraphPad Software Inc., La Jolla, CA). Differences were considered to be significant when *P* < 0.05.

## Results

### Vγ9Vδ2-T cells selectively target malignant cells in the absence of exogenous pamidronate in 2D

**Figure 2:**
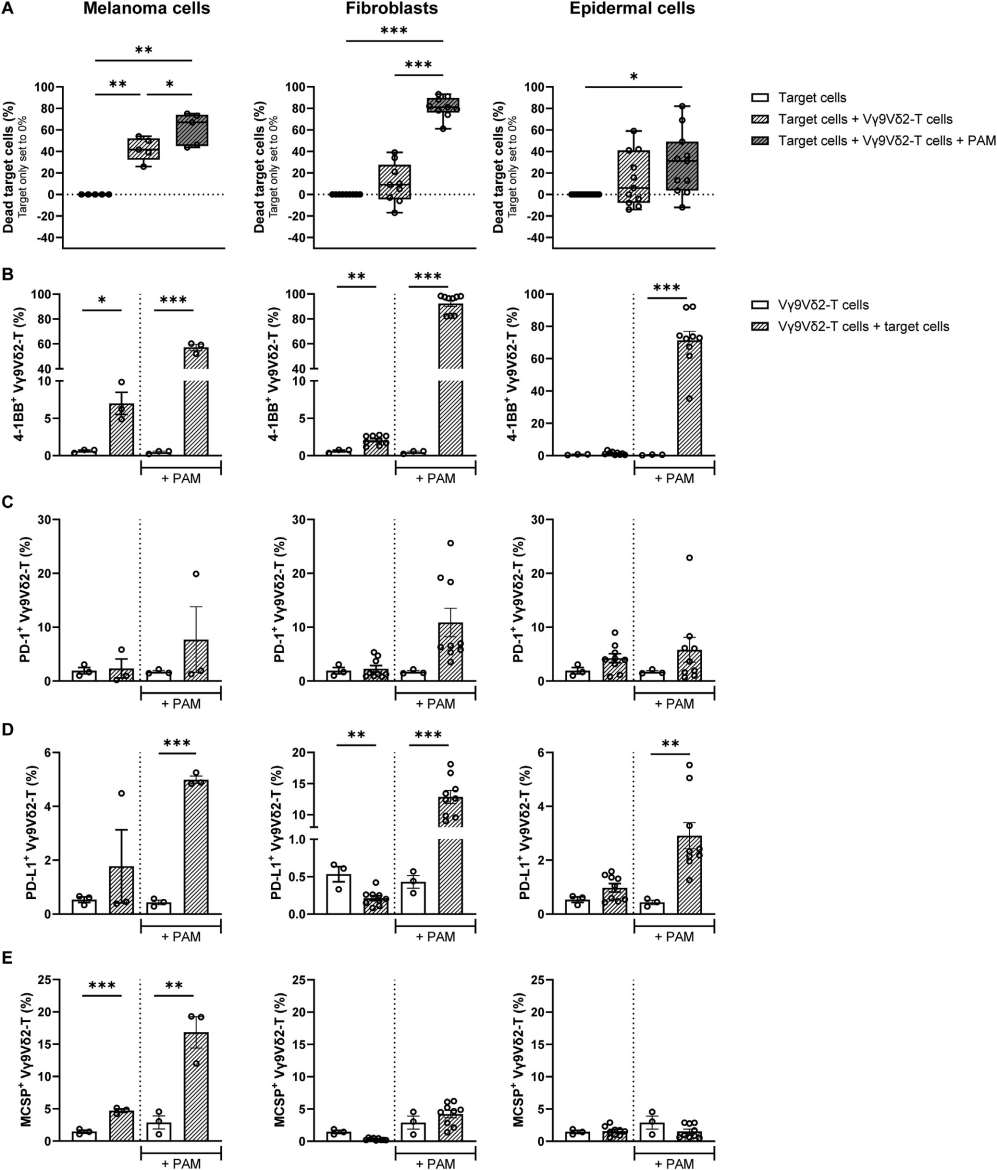
**Vγ9Vδ2-T cell-mediated killing of malignant melanoma cells and normal human skin cells and subsequent activation in the presence or absence of pamidronate (PAM).** Vγ9Vδ2-T cells were co-cultured for 24 h with either A375 melanoma cells, fibroblasts, or epidermal cells at an effector-to-target (E-to-T) ratio of 1 : 1 in the absence or presence of 10 μM PAM. (A) Vγ9Vδ2-T cell-induced cell death of melanoma cells, fibroblasts, or epidermal cells in the absence (white line pattern boxes) or presence (gray line pattern boxes) of PAM. One-way analysis of variance (^∗^*P* < 0.05, ^∗∗^*P* < 0.01, and ^∗∗∗^*P* < 0.0001). Expression of (B) 4-1BB, (C) programmed cell death protein-1 (PD-1), and (D) programmed death-ligand 1 (PD-L1) on the Vγ9Vδ2-T cells at baseline (white bars) or after co-culture with A375 melanoma cells, fibroblasts, or epidermal cells (line pattern bars) in the absence or presence (+PAM) of PAM. Unpaired *t*-test (^∗^*P* < 0.05, ^∗∗^*P* < 0.01, and ^∗∗∗^*P* < 0.0001). (E) Melanoma-associated chondroitin sulfate proteoglycan (MCSP) uptake by Vγ9Vδ2-T cells at baseline (white bars) or after co-culture with A375 melanoma cells, fibroblasts, or epidermal cells (line pattern bars) in the absence or presence (+ PAM) of PAM. (A–D) Vγ9Vδ2-T cells were isolated from three or more healthy donors. Fibroblasts and epidermal cells were isolated from 3 and 4 healthy donors, respectively. Each circle indicates an independent experiment where a different Vγ9Vδ2-T cell donor and/or a different skin cell donor were used. Data from *n* ≥ 3 independent experiments are shown as mean ± standard error of the mean. Unpaired *t*-test (^∗∗^*P* < 0.01).

The ability of Vγ9Vδ2-T cells to selectively kill human melanoma cells was first tested in cell suspension co-cultures. Beside the melanoma cells, normal skin cells (primary fibroblasts and epidermal cells) were co-cultured in 2D for 24 h with Vγ9Vδ2-T cells. While marginal target cell death could be observed for fibroblasts (mean: 10.22%) and epidermal cells (mean: 13.83%), ∼42% of melanoma cells were lysed upon co-culture with Vγ9Vδ2-T cells (Figure 2A), indicating that Vγ9Vδ2-T cells selectively target malignant cells in the absence of additional stimuli. Consistent with this, activation of Vγ9Vδ2-T cells was most predominant after co-culture with melanoma cells, as seen by the increased expression of the surface marker 4-1BB (Figure 2B). Of note, fibroblasts were also able to induce higher 4-1BB levels on Vγ9Vδ2-T cells, compared to the Vγ9Vδ2-T cell alone condition, although to a lesser extent than melanoma cells. Almost no differences in PD-1 and PD-L1 expression could be found on Vγ9Vδ2-T cells after their co-culture with either melanoma or healthy skin cells (Figure 2C and D). PD-L1 expression actually decreased on Vγ9Vδ2-T cells upon their co-culture with fibroblasts (Figure 2D).

Recognition of malignant cells by Vγ9Vδ2-T cells happens via elevated levels of pAg molecules, which induce a conformational change in butyrophilin 3A1 (BTN3A1), which is in turn recognized in a complex with BTN2A1 by Vγ9Vδ2-T cells through cognate interaction with their TCR.^20^^,^^21^ Levels of pAgs can be increased under stress by aminobisphosphonates, such as PAM,^2^ leading to increased Vγ9Vδ2-T cell-mediated antitumor activity.^22^ Although lytic activity against the melanoma cells indeed went up when PAM was added to the co-cultures, Vγ9Vδ2-T cells failed to maintain their tumor specificity and now also induced healthy skin cell lysis (mean: 61.20% for melanoma cells, 80.78% for fibroblasts, and 29.08% for epidermal cells; Figure 2A). Vγ9Vδ2-T cell activation in the presence of PAM could be observed in all three co-culture conditions, with fibroblasts inducing the most profound activation, resulting in almost all Vγ9Vδ2-T cells to express 4-1BB (mean: 92.40%; Figure 2B). Consistent with this result, BTN3A was expressed by virtually all melanoma cells as well as fibroblasts and epidermal cells, with even higher per-cell expression levels on the latter two, regardless of the absence or presence of exogenously added PAM (Supplementary Figure S1, available at https://doi.org/10.1016/j.iotech.2024.100724).

**Figure 3:**
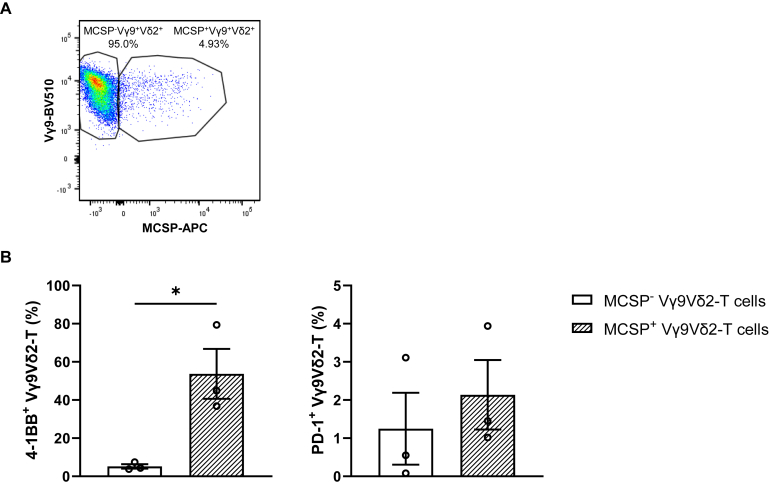
**Melanoma-associated chondroitin sulfate proteoglycan-positive (MCSP**^**+**^**) Vγ9Vδ2-T cells are more activated than their MCSP**^**−**^**counterpart.** Vγ9Vδ2-T cells were co-cultured for 24 h with A375 melanoma cells at an effector-to-target (E-to-T) ratio of 1 : 1 in the absence of pamidronate (PAM). (A) Representative fluorescence-activated cell sorting (FACS) plot of MCSP uptake by the Vγ9Vδ2-T cells. (B) Expression of the surface markers 4-1BB and programmed cell death protein-1 on MCSP^−^ (white bars) and MCSP^+^ (line pattern bars) Vγ9Vδ2-T cells. Vγ9Vδ2-T cells were isolated from three healthy donors. Each symbol indicates an independent experiment in which a different Vγ9Vδ2-T cell donor was used. Data from *n* = 3 independent experiments are shown as mean ± standard error of the mean. Unpaired *t*-test (^∗^*P* < 0.05).

Following the establishment of an immunological synapse between a T lymphocyte and a target cell, membrane patches from the target cells are transferred to the T cell via a mechanism known as trogocytosis.^23^^,^^24^ Membrane-expressed MCSP, a widely expressed molecule at the cell surface of melanoma cells, on T cells was previously shown to be related to both their tumor specificity and cytolytic activity in patients with melanoma.^24^^,^^25^ In line with this, we found MCSP uptake by the Vγ9Vδ2-T cells to be restricted to co-cultures with melanoma cells and to be increased upon addition of PAM (Figure 2E). A representative fluorescence-activated cell sorting (FACS) dot-plot example of MCSP uptake by the Vγ9Vδ2-T cells is shown in Figure 3A. Of note, those Vγ9Vδ2-T cells, which acquired MCSP from the melanoma cells, were also more active compared with their MCSP^−^ counterpart, as shown by higher expression of 4-1BB (5% MCSP^−^4-1BB^+^ *versus* 54% MCSP^+^4-1BB^+^ Vγ9Vδ2-T cells; Figure 3B). Although at low levels, a similar nonsignificant trend was observed for PD-1.

In view of the observed loss of tumor-selective kill by the Vγ9Vδ2-T cells upon addition of PAM, we decided to next test their functionality in the previously described 3D Mel-RhS^16^ in the absence of PAM.

### Vγ9Vδ2-T cells survive in the *in vitro* 3D skin and melanoma microenvironment

Vγ9Vδ2-T cells were introduced into Mel-RhS or control RhS via injection and cultured within this 3D microenvironment for 1-3 days. Figure 1B presents a schematic overview of the injection procedure and downstream analyses of the Vγ9Vδ2-T cell-complemented RhS and Mel-RhS.

**Figure 4:**
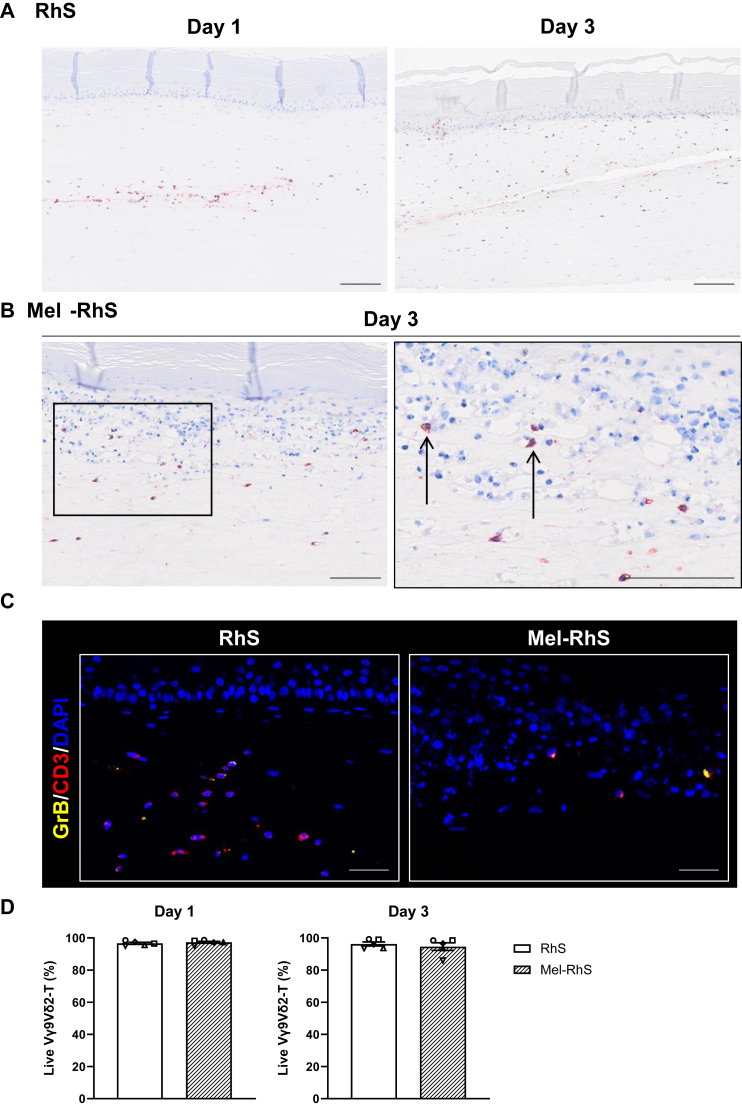
**Vγ9Vδ2-T cells injected into RhS and Mel-RhS are viable up to 3 days in the 3D model.** (A) CD3^+^ Vγ9Vδ2-T cells in the RhS model. An example of injection point/needle track in RhS is displayed after 1 *versus* 3 days after injection. Scale bar = 100 μm. (B) In Mel-RhS, a fraction of Vγ9Vδ2-T cells was also found within the melanoma nests by day 3, shown in the right picture at higher magnification (black arrows). Scale bar = 100 μm. (C) Vγ9Vδ2-T cells retain their functional innate immune cell-like phenotype in the 3D microenvironment as shown 3 days after injection by immunofluorescent staining of granzyme B (GrB; yellow) combined with CD3 (red; Vγ9Vδ2-T cells) and 4′,6-diamidino-2-phenylindole (DAPI; blue; nuclei). Scale bar = 20 μm. (D) Viability of Vγ9Vδ2-T cells 1 or 3 days after their integration in RhS (white bars) and Mel-RhS (line pattern bars) without the preincubation with pamidronate. Vγ9Vδ2-T cells were isolated from five healthy donors. Skin cells were isolated from three healthy donors. Each symbol indicates an independent experiment in which a different Vγ9Vδ2-T cell donor and/or a different skin cell donor were used. Data from *n* = 5 independent experiments are shown as mean ± standard error of the mean.

Vγ9Vδ2-T cells were visualized in the RhS and Mel-RhS models via CD3 staining. While Vγ9Vδ2-T cells seemed to localize closer to the injection site at day 1, they had migrated and were spread throughout the dermal compartment by day 3, with also clear infiltration into the basal layers of the epidermis (Figure 4A). In the Mel-RhS, a fraction of Vγ9Vδ2-T cells could also be found in proximity of or within the outer edge of the tumor nests (Figure 4B). Their expression of granzyme B in both RhS and Mel-RhS further showed them to have retained their innate cytolytic potential (Figure 4C). Throughout this 3-day culture the Vγ9Vδ2-T cells retained their viability as shown by flow cytometry after enzymatic dissociation of the RhS and Mel-RhS to single-cell suspensions (Figure 4D).

### Vγ9Vδ2-T cells are active within the 3D microenvironment

**Figure 5:**
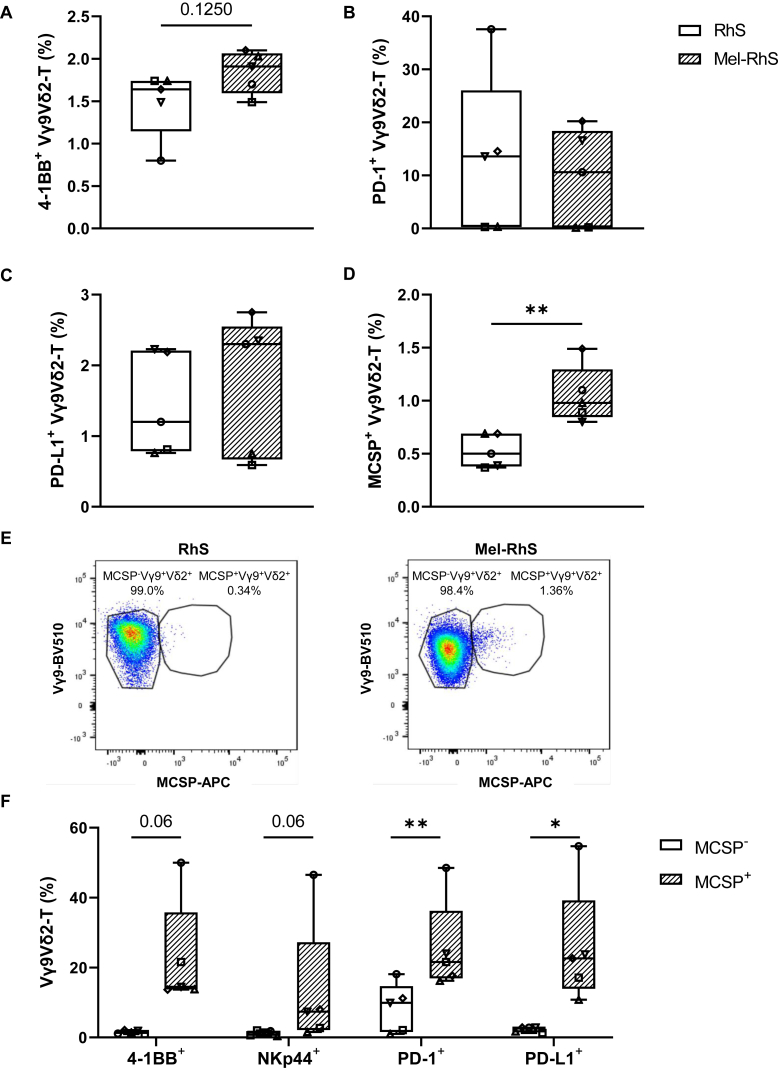
**Elevated activation status of melanoma-decorated Vγ9Vδ2-T cells 3 days after their injection into Mel-RhS.** Vγ9Vδ2-T cells were isolated from dissociated RhS (white boxes) and Mel-RhS (line pattern boxes) and assessed for the expression of the surface markers (A) 4-1BB, (B) programmed cell death protein-1 (PD-1), and (C) programmed death-ligand 1 (PD-L1), and for (D) melanoma-associated chondroitin sulfate proteoglycan (MCSP) uptake. (E) Representative fluorescence-activated cell sorting (FACS) plots of MCSP uptake by the Vγ9Vδ2-T cells and gating of MCSP-decorated Vγ9Vδ2-T cells 3 days after injection in RhS or Mel-RhS. (F) Expression of the surface markers 4-1BB, NKp44, PD-1, and PD-L1 on MCSP^−^ (white boxes) versus MCSP^+^ Vγ9Vδ2-T cells (line pattern boxes), isolated from Mel-RhS 3 days after injection. (A–D and F) Vγ9Vδ2-T cells were isolated from five healthy donors. Skin cells were isolated from three healthy donors. Each symbol indicates an independent experiment where a different Vγ9Vδ2-T cell donor and/or a different skin cell donor were used. Data from *n* = 5 independent experiments are shown as min to max box plots. Paired *t*-test (^∗^*P* < 0.05 and ^∗∗^*P* < 0.01). NK, natural killer.

Activation status of the Vγ9Vδ2-T cells retrieved from the 3-day-cultured RhS and Mel-RhS was investigated by means of 4-1BB, PD-1, and PD-L1 expression (Figure 5A-C). Albeit not significant, increased expression of 4-1BB was found on Mel-RhS-isolated Vγ9Vδ2-T cells, compared with those isolated from RhS, whereas no PD-1 or PD-L1 upregulation was detected (Figure 5B and C). These observations were consistent with findings from the 2D co-cultures in the absence of PAM (Figure 2C and D). In line with the observation of melanoma infiltration by day 3 of culture, we also found clear MCSP expression on the cell surface of a small fraction of Mel-RhS-isolated Vγ9Vδ2-T cells, which likely acquired the melanoma-associated antigen via trogocytosis^26^^,^^27^ (Figure 5D). Upon gating of the MCSP^−^ and MCSP^+^ fractions (Figure 5E), the latter was found to be selectively activated, as determined by (near-)significantly elevated levels of 4-1BB, NKp44, PD-1, and PD-L1, likely due to cognate interaction with the melanoma cells (Figure 5F).

### Extra stimuli might be necessary to increase the activation status of Vγ9Vδ2-T cells within the 3D microenvironment

**Figure 6:**
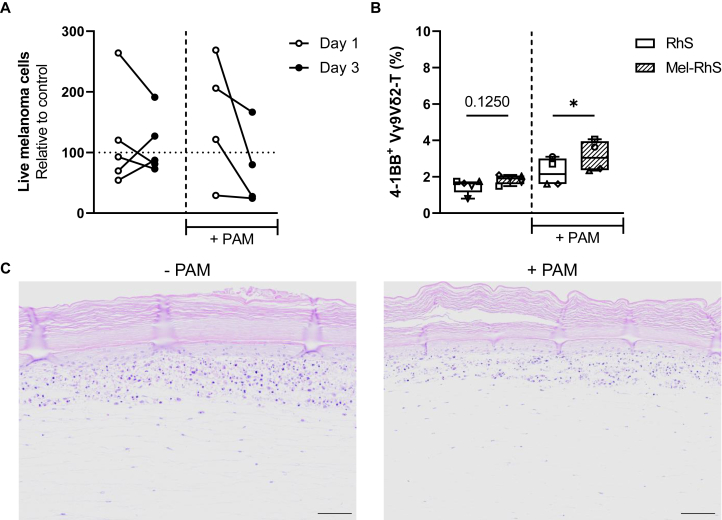
**Incubation of Mel-RhS with pamidronate (PAM) increases oncolytic activity and activation status of Vγ9Vδ2-T cells.** (A) Melanoma cells were isolated from Mel-RhS, which were preincubated in the absence or presence (+ PAM) of PAM, 1 or 3 days after Vγ9Vδ2-T cell injection. Their viability was assessed via fluorescence-activated cell sorting (FACS) and normalized to a Mel-RhS control (mock injection), set as 100% melanoma cell viability (dotted line). Vγ9Vδ2-T cells were isolated from five or four (+ PAM) healthy donors. Skin cells were isolated from three or two (+ PAM) healthy donors. Each circle indicates an independent experiment where a different Vγ9Vδ2-T cell donor and/or a different skin cell donor were used. Data from *n* ≥ 4 independent experiments is shown. (B) Expression of the surface marker 4-1BB on Vγ9Vδ2-T cells isolated from RhS (white boxes) or Mel-RhS (line pattern boxes), which were preincubated in the absence or presence (+ PAM) of PAM, 3 days after injection. Vγ9Vδ2-T cells were isolated from five or four (+ PAM) healthy donors. Skin cells were isolated from three or two (+ PAM) healthy donors. Each symbol indicates an independent experiment where a different Vγ9Vδ2-T cell donor and/or a different skin cell donor were used. Data from *n* ≥ 4 independent experiments are shown as min to max box plots. Paired *t*-test (^∗^*P* < 0.05). (C) Hematoxylin and eosin staining of Mel-RhS cultured in the absence (− PAM) or presence (+ PAM) of PAM 3 days after Vγ9Vδ2-T cell injection. Scale bar = 100 μm.

As Vγ9Vδ2-T cells in the Mel-RhS had reached the tumor nests at some point by day 3 of culture, we reasoned that any oncolytic activity should result in a reduction of absolute melanoma cell numbers from day 1 to 3. We did not find consistent evidence of this unless we carried out a (pre)incubation of Mel-RhS with PAM (Figure 6A). Although not significant, due to considerable variability in the absolute numbers of melanoma cells retrieved from the Mel-RhS, we did find evidence of oncolytic activity upon PAM conditioning. Addition of PAM also increased the activation status of the Vγ9Vδ2-T cells, as shown by increased 4-1BB expression (Figure 6B). Although data from our 2D co-cultures indicated a loss of tumor-selective lysis induced by Vγ9Vδ2-T cells in the presence of PAM (Figure 2A), we found no evidence of loss of integrity of the skin tissue structure in Mel-RhS complemented with Vγ9Vδ2-T cells and incubated with PAM (Figure 6C).

## Discussion

In this study, we aimed to showcase the utility of the previously described Mel-RhS^15^^,^^16^ as a viable platform for testing T cell-based immunotherapies, while simultaneously characterizing the response of Vγ9Vδ2-T cells against melanoma in a human 3D *in vitro* setting. Vγ9Vδ2-T cells were an attractive choice to test in this model because of their HLA-unrestricted mode of action, being at the crossroads of adaptive and innate immunity, while their importance in antitumor immunity with prognostic significance had been amply demonstrated. Deconvolution studies identified tumor-infiltrating γδ-T cells as the most favorable subset in terms of prognosis across all human tumor types.^28^ The prognostic value of Vγ9Vδ2-T cells specifically was shown to be less uniform and more context dependent.^9^^,^^29^, ^30^, ^31^

We were able to show the ability of Vγ9Vδ2-T cells to migrate toward and infiltrate into melanoma nests in the 3D skin microenvironment over the course of 3 days, an important characteristic and precondition for Vγ9Vδ2-T cell-based immunotherapies to be effective. Subsequent cognate interaction with the melanoma cells was evidenced by specific activation of the Vγ9Vδ2-T cells with apparent melanoma trogocytosis (*i.e.* MCSP expression). Previous studies showed trogocytosis to be a TCR-mediated process, to occur during T cell-mediated oncolysis, and to be directly related to the oncolytic ability of T cell clones.^24^^,^^25^ In our Mel-RhS model, the limited proportion of MCSP^+^ cells appeared to mirror the restricted presence of Vγ9Vδ2-T cells infiltrating into the tumor nests, as detected in tissue sections by immunohistochemistry. This plausibly explains the absence of activation marker upregulation when examining the overall Vγ9Vδ2-T cell population and also the lack of evidence of consistent melanoma cell kill, assessed by reduced melanoma cell numbers between day 1 and 3 of culture. TCR-mediated activation of Vγ9Vδ2-T cells depends on the recognition of conformational changes in BTN3A1, induced by pAgs, which are elevated in tumors due to dysregulation of the mevalonate pathway.^2^^,^^32^ We found that PAM-induced pAg upregulation was needed in the Mel-RhS to obtain evidence of effective oncolysis. This demonstrates the intrinsic capability of Vγ9Vδ2-T cells to lyse the tumor cells, but points to the possible need for their increased cognate binding to the tumor targets. In 2D cell suspension co-cultures, we found PAM to induce Vγ9Vδ2-T cell-mediated killing of healthy BTN3A^+^ fibroblasts and keratinocytes, implying an increased risk of on-target, off-tumor toxicity. Of note, we found no evidence of this in the 3D model, possibly pointing to a differential pAg induction or BTN3A expression in that context. Nevertheless, perhaps a more specific approach such as bispecific Vγ9Vδ2-T cell engagers would provide a safe and effective alternative. Indeed, such engagers have shown promise in redirecting the cytotoxic activity of CD3^+^ T cells toward tumors, making them a highly viable immunotherapeutic strategy for both hematological and solid malignancies.^33^, ^34^, ^35^, ^36^ The Vγ9Vδ2-T cell-complemented Mel-RhS model would provide an attractive platform to test their efficacy in a 3D tissue context.

A limitation of this study is the use of a single melanoma cell line (*i.e.* A375). This was due to the complex and time-consuming nature of the Mel-RhS. A375 previously exhibited a mediocre capacity to induce CXCL10 in the investigated 3D *in vitro* model.^16^ Therefore considering the key role CXCL10 plays in governing the recruitment of T cells into the tumor microenvironment,^37^ the use of alternative cell lines resulting in higher CXCL10 levels should and will be further explored in follow-up studies. Alternatively, the quantity of injected Vγ9Vδ2-T cells could be increased. However, it is worth noting that the standardization/automation of the injection procedure (e.g. by microneedle patches) may also contribute to an improved infiltration rate.

Incorporation of immune cells into 3D *in vitro* tumor models remains challenging and has not been extensively investigated.^38^ To the best of our knowledge, the present study reports for the first time the inclusion of Vγ9Vδ2-T cells in a Mel-RhS and contributes to the currently very limited existing literature showcasing the feasibility of integrating immune cells into complex 3D tumor models.^13^^,^^14^ For instance, while the generation of DC-complemented RhS has been achieved by various research groups,^39^, ^40^, ^41^, ^42^, ^43^, ^44^ to date, only one study, conducted in 2020, reported the injection into a human organotypic skin melanoma culture of type-2 conventional DCs (cDC2s), which were observed to remain viable for 2 days.^14^ In contrast to alternative approaches, such as seeding the immune cells beneath the 3D model, this technique enabled direct integration of cDC2s into the 3D microenvironment. Consequently, a similar approach was adopted in this study. While here we report survival and maintained viability of the injected Vγ9Vδ2-T cells up to 3 days after injection, preliminary evidence suggests the potential extension of this time frame to 7 days (unpublished data).

### Conclusions

This study provides valuable insights into the behavior and potential applications of Vγ9Vδ2-T cells in cancer research. Our findings highlight the viability and persistence of Vγ9Vδ2-T cells within the 3D microenvironment (and thus the ability of the developed Mel-RhS to support survival of the employed T cell subset), their migratory and antitumor functionality, and the suitability of the model for testing immune cell-based therapies, contributing to the understanding of Vγ9Vδ2-T cell biology and their potential as an effective immunotherapeutic strategy for melanoma treatment. Moreover, and importantly, the currently presented model may also serve to study generally applicable effector T cell functions in a human 3D tumor tissue setting.
