# Supplementary material for: An organotypic human melanoma-in-skin model as an in vitro tool for testing Vγ9Vδ2-T cell-based immunotherapy
Source: Immunooncol Technol. 2024 Jul 10;24:100724. doi: 10.1016/j.iotech.2024.100724 (PMC11363583; doi:10.1016/j.iotech.2024.100724)
Supplement: Supplementary Figure S1 [file mmc1.pptx]

## Slide 1
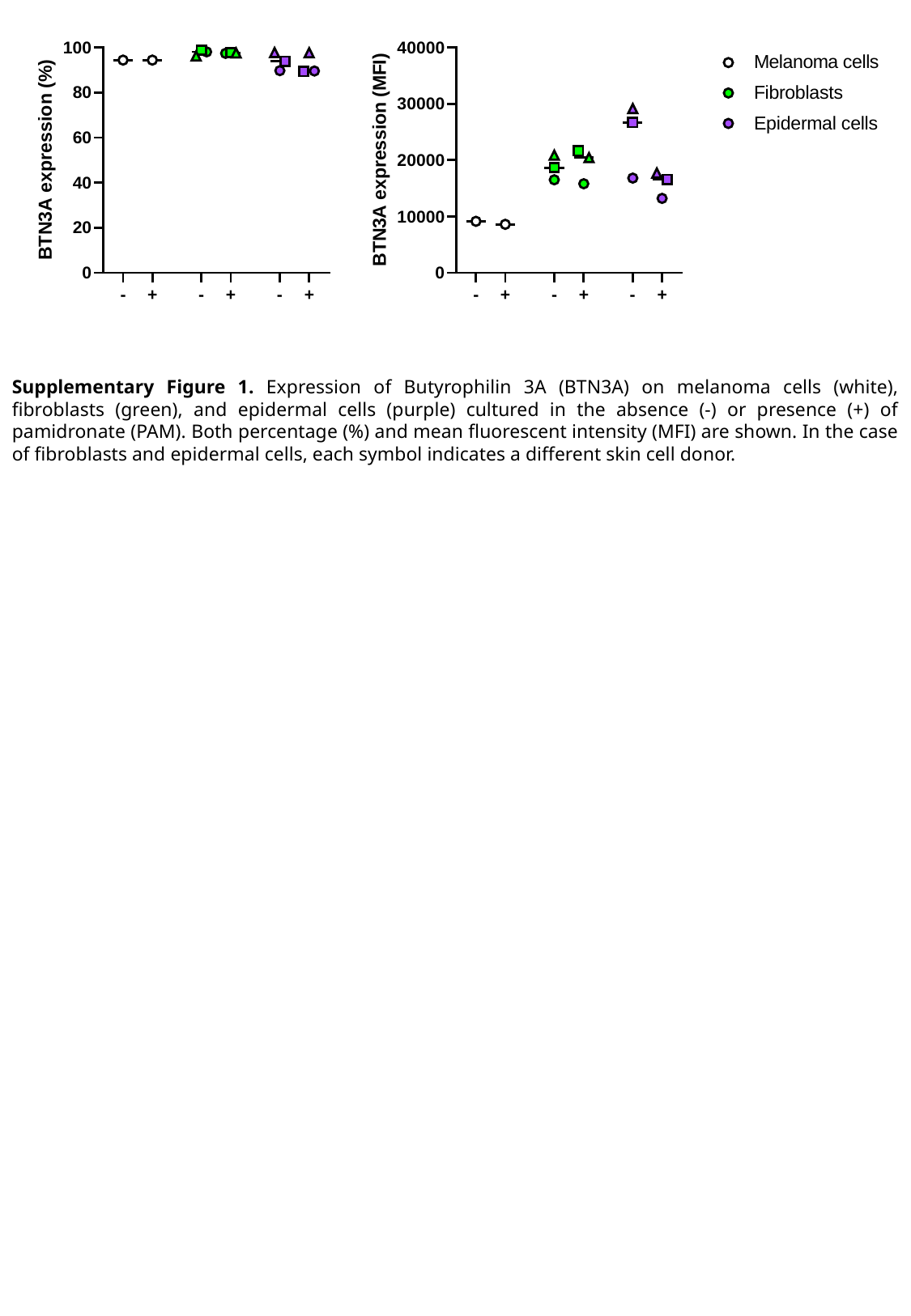

Supplementary Figure 1. Expression of Butyrophilin 3A (BTN3A) on melanoma cells (white), fibroblasts (green), and epidermal cells (purple) cultured in the absence (-) or presence (+) of pamidronate (PAM). Both percentage (%) and mean fluorescent intensity (MFI) are shown. In the case of fibroblasts and epidermal cells, each symbol indicates a different skin cell donor.
